# Supplementary material for: Empirical methods for controlling false positives and estimating confidence in ChIP-Seq peaks
Source: BMC Bioinformatics. 2008 Dec 5;9:523. doi: 10.1186/1471-2105-9-523 (PMC2628906; doi:10.1186/1471-2105-9-523)
Supplement: Additional File 1 — A variety of html documents from the USeq web site detailing the available applications, their best usage, output file type descriptions, command line menus, etc. [file 1471-2105-9-523-S1.zip › USeqUserGuides/JavaDoc.zip!/JavaDoc/trans/tpmap/WindowMaker.html]

WindowMaker


|  |  |  |
| --- | --- | --- |
| **Overview** \| **Package** \| **Class** \| **Use** \| **Tree** \| **Deprecated** \| **Index** \| **Help** | |  |
| **PREV CLASS**   NEXT CLASS | **FRAMES**    **NO FRAMES**     **All Classes** |  |
| SUMMARY: NESTED \| FIELD \| CONSTR \| METHOD | DETAIL: FIELD \| CONSTR \| METHOD |  |


---


## trans.tpmap Class WindowMaker

```
java.lang.Object
  trans.tpmap.WindowMaker
```

---

``` public class WindowMaker extends java.lang.Object ```

Creates Windows (start position, end position) of a given length, containing a given number of start positions and maximum number of features.
Remember for any given position it may have multiple features, ie spotted multiple times on the array due to multiple hits.
Thus a window with 3 positions, each with 10 spottings has 30 features for the window. Can limit the amount added by setting the
maxNumFeatures field, it defaults to 1,000,000,000.
Used to assemble subsets of the array data for performing statistical tests. Each window contains a unique set
of features, tries to maximize its size to the max size, and is only saved if it contains the minimum number of
features.

---

**Constructor Summary**

`WindowMaker(int windowSize,
int minNumOligos)`

`WindowMaker(int windowSize,
int minNumOligos,
int maxNumFeatures)`


| **Method Summary** | |
| --- | --- |
| `static java.lang.String[]` | `extractChrFiles(java.io.File directory)` |
| `int` | `getMaxNumFeatures()` |
| `static void` | `main(java.lang.String[] args)` |
| `int[][]` | `makeWindows(int[] bpStartPositions)`             Takes an int[] of sorted bp start positions and finds unique windows of a maximized width with a minimal # of features. |
| `void` | `makeWindowsFromFiles(java.lang.String[] files)`             Takes int[] files containing bp start positions for each feature, ordered. |
| `static int` | `numberUniqueValues(int[] values, int startIndex, int stopIndex)`             Given a sorted set of values, a start and stop index (inclusive) returns the number of unique values. |
| `void` | `setMaxNumFeatures(int maxNumFeatures)` |

**Methods inherited from class java.lang.Object**

`equals, getClass, hashCode, notify, notifyAll, toString, wait, wait, wait`

**Constructor Detail**

### WindowMaker

```
public WindowMaker(int windowSize,
                   int minNumOligos)
```

---


### WindowMaker

```
public WindowMaker(int windowSize,
                   int minNumOligos,
                   int maxNumFeatures)
```


**Method Detail**

### makeWindowsFromFiles

```
public void makeWindowsFromFiles(java.lang.String[] files)
```

:   Takes int[] files containing bp start positions for each feature, ordered. Saves the results as a
    int[][] where each window has a start index and stop index that correspond to the indexes in the original
    bp start positions. Indexes of indexes...

---


### numberUniqueValues

```
public static int numberUniqueValues(int[] values,
                                     int startIndex,
                                     int stopIndex)
```

:   Given a sorted set of values, a start and stop index (inclusive) returns the number of unique values.

---


### makeWindows

```
public int[][] makeWindows(int[] bpStartPositions)
```

:   Takes an int[] of sorted bp start positions and finds unique windows of
    a maximized width with a minimal # of features. Returns
    an int[window number][start index, stop index]. Stop is inclusive!

---


### extractChrFiles

```
public static java.lang.String[] extractChrFiles(java.io.File directory)
```

---


### getMaxNumFeatures

```
public int getMaxNumFeatures()
```

---


### setMaxNumFeatures

```
public void setMaxNumFeatures(int maxNumFeatures)
```

---


### main

```
public static void main(java.lang.String[] args)
```


---


|  |  |  |
| --- | --- | --- |
| **Overview** \| **Package** \| **Class** \| **Use** \| **Tree** \| **Deprecated** \| **Index** \| **Help** | |  |
| **PREV CLASS**   NEXT CLASS | **FRAMES**    **NO FRAMES**     **All Classes** |  |
| SUMMARY: NESTED \| FIELD \| CONSTR \| METHOD | DETAIL: FIELD \| CONSTR \| METHOD |  |


---
